# Assessment of a Colonoscopy Triage Sheet for Use in a Province-Wide Population-Based Colorectal Screening Program

**DOI:** 10.1155/2016/4712192

**Published:** 2016-07-03

**Authors:** Nour Sharara, Sabrina Nolan, Maida Sewitch, Myriam Martel, Maria Dias, Alan N. Barkun

**Affiliations:** ^1^Division of Gastroenterology, McGill University Health Centre, McGill University, 1650 Cedar Avenue, Room D7-346, Montreal, QC, Canada H3G 1A4; ^2^Division of Clinical Epidemiology, Research Institute, McGill University Health Centre, McGill University, 1650 Cedar Avenue, Room D7-346, Montreal, QC, Canada H3G 1A4; ^3^Department of Nursing, McGill University Health Centre, McGill University, 1650 Cedar Avenue, Room D7-346, Montreal, QC, Canada H3G 1A4

## Abstract

*Background and Aims*. A colonoscopy triage sheet (CTS) integrating 6 hierarchical scheduling priorities based on indications for screening, surveillance, or symptoms was designed for colonoscopy referral. We compared CTS priority ratings by referring physicians and endoscopists, assessing yields.* Methods*. Retrospective study of consecutive patients. Data were collected on demographics, CTS and endoscopist priority ratings, and endoscopic findings. Weighted kappa values measured interrater agreement on priority assignment. Predictors of agreement and lesions were identified using multivariable analysis.* Results*. Among 1230 patients (60.3 years, 52.5% female), clinically significant lesions included tumors (1.1%), polyps per patient ≥ 10 mm (7.6%), and ileocolitis (4.6%). Moderate agreement was found between referring physician and endoscopist on all 6 priorities (weighted kappa 0.55 (0.51; 0.59)). P4 and P5 ratings predicted increased agreement (range of OR for P4: 2.47–4.57; P5: 1.58–2.93). Predictors of clinically significant findings were male gender (OR 1.44, 1.03–2.03) and P1/P2 priorities that were significantly superior to P3 (OR = 2.14; 1.04–4.43), P4 (OR = 2.90; 1.35–6.23), and P5 (OR = 4.30; 2.08–8.88).* Conclusion*. Priority-assignment agreement is moderate and highest for less urgent ratings. Predictors of clinically significant findings validate the hierarchal priority scheme. Broader validation and physician education are needed.

## 1. Introduction

Colorectal cancer (CRC) is a major cause of death worldwide. Colorectal cancer is the third most commonly diagnosed cancer in Canada (excluding nonmelanoma skin cancers) and it is the second leading cause of death from cancer in men and the third leading cause of death from cancer in women in Canada [1]. Endoscopic resources are, however, in most regions of Canada limited with both selected screening (positive FOBT or imaging) and symptomatic patients requiring a timely colonoscopy.

Amidst long delays in access to health care services in Canada, the Canadian Association of Gastroenterology (CAG) developed evidence-based recommendations for appropriate maximal wait times for colonoscopies in order to promote the efficient and equitable use of endoscopic resources [2].

Based on the CAG guidelines and in the context of an Open-Access Endoscopy system (without a prior clinic consultation with the endoscopist), a 1-page colonoscopy triage sheet (CTS) was developed for the entire province to improve the quality, efficiency, and equitable delivery of all colonoscopy services. The CTS captures all pertinent clinical information (symptoms or screening/surveillance indication) and medical history as recorded by the referring physician, as well as a section that allows flagging patients who require a prior consultation in person because of existing comorbidities (e.g., need for anticoagulation).

Prior to province-wide implementation, we piloted the use of the CTS in one academic medical centre (the Montreal General Hospital (MGH) site of the McGill University Health Centre (MUHC)).

We sought (1) to compare the priority and indication selected by the referring physician on the CTS with the ones additionally determined by the endoscopist on the day of the open-access colonoscopy in order to measure the agreement between physicians and (2) to assess the yield of the different CTS priorities in order to evaluate the yield of priorities and indications with regard to colonoscopy findings.

## 2. Methods

### 2.1. The Colonoscopy Triage Sheet (CTS)

**Figure 1:**
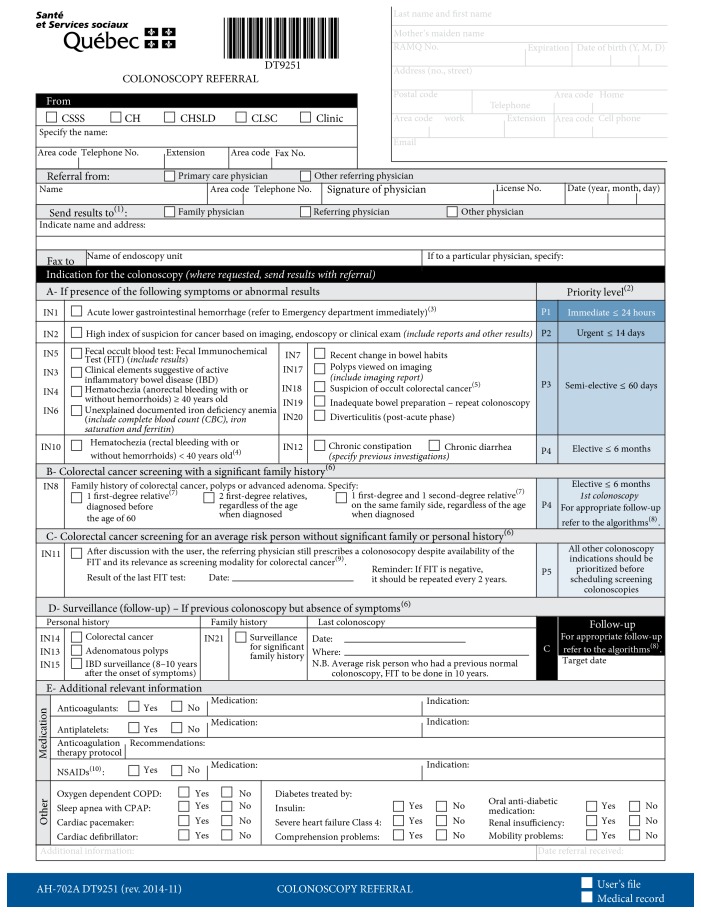
Colonoscopy triage sheet (CTS). In this later version of the CTS, IN8, IN9, and IN16 have been regrouped as IN8. If more than one indication is written on the colonoscopy referral form, the indication with the highest level of priority will be used for the colonoscopy.

The CTS (Figure 1) was developed by the Department of Nursing and the Division of Gastroenterology at the MUHC in conjunction with the Quebec Ministry of Health based on an extensive review of the literature and available wait times recommendations, including those issued by the CAG in 2006 [2]. The CTS was further refined and endorsed by multiple user groups, including Quebec medical specialty associations, namely, the AGEQ (Association des Gastro-Entérologues du Québec), the AQC (Association Québecoise de Chirurgie), the ASMIQ (Association des Spécialistes en Médecine Interne du Québec), primary care physicians associations (FMOQ: Fédération des Médecins Omnipraticiens du Québec), and the Quebec Ministry of Health (MSSS).

The CTS lists a hierarchy of 19 colonoscopy indications related to screening, surveillance, or symptoms that are grouped according to 6 scheduling priorities (e.g., 24 hours to 2 weeks, 2 months, and 6 months or more, Figure 1). In this hierarchal listing, patients with symptoms are categorized into priorities P1 to P4 (in decreasing urgency), patients requiring a colonoscopy for average-risk screening are assigned to P5, and those undergoing a colonoscopy for surveillance following previous colonoscopy are assigned to P6.

*Notes on CTS*. In Figure 1, (1) a copy of the results must be sent to the referring physician; (2) the proposed timelines and priorities are targets for improvement to be achieved and not clinical practice directives; the referring physician can communicate with the endoscopist if needed; (3) definition of acute lower gastrointestinal hemorrhage is provided: hematochezia and hemodynamic instability, important drop in hemoglobin values, and/or need for blood transfusions; (4) sigmoidoscopy is also indicated as a diagnostic exam in this situation; (5) paraneoplastic syndrome is mentioned; (6) if the user complains of new onset of symptoms, it is the responsibility of the referring physician to do the appropriate follow-up and to notify the endoscopist to whom the referral was initially sent; (7) definition of (i) first-degree relative, father/mother, brother/sister, or child, and (ii) second-degree relative, grandparent, uncle/aunt, and nephew/niece, is provided; (8) the algorithms are available: http://www.msss.gouv.qc.ca/professionnels/pqdccr/; (9) the recommended screening test for an average-risk person (50–74 years old, asymptomatic, without any family or personal colorectal cancer or adenomatous polyp history) is the fecal occult blood test: Fecal Immunochemical Test (FIT); the colonoscopy is prescribed to confirm the diagnosis when a FIT is positive (IN5); (10) it is not necessary to stop aspirin, Persantine, or Aggrenox before a colonoscopy.

### 2.2. Study Design and Population

A retrospective study was conducted among outpatients referred to the MGH site for colonoscopy which included all possible referring physicians to the institution. Only gastrointestinal endoscopists performed the study colonoscopies. Outpatients who had undergone a colonoscopy between January 1, 2013, and July 31, 2013, having a CTS completed by the referring physician, were included. Only the first colonoscopy for a given patient was included, if more than one was performed during the study period. The referring physician sent the CTS to the MGH by fax/mail and the CTS data were entered by a secretary into the institutional scheduling software as part of a standardized, validated administrative process (Medivisit®, NYC, NY, USA). CTS data included the indication reported by the referring physician and the corresponding priority and the assigned target date of the procedure by the endoscopy unit. On the day of the colonoscopy, after taking a brief history and prior to performing the colonoscopy, the endoscopist entered pertinent clinical and endoscopic data into an endoscopy reporting software (EndoWorks®, Central Valley, PA, USA), including the indication in the opinion of the endoscopist. The endoscopists were familiar with the CTS but were not asked to prioritize the indication since every indication corresponds to only one possible priority rating. An independent blinded research assistant assigned the corresponding priority using the CTS form.

Demographic data were compiled on all the referring physicians who sent the CTS included in the analysis and on all endoscopists who performed colonoscopies on the study population.

### 2.3. Data Collection

Patients were identified by electronic search of the two institutional scheduling and endoscopic reporting software programs. Trained personnel extracted and entered data into a dedicated electronic form, using standardized methodology. The data included the date of the referral request, coded identity of the referring physician (including GP/specialty), the priority assigned according to the indication of the referral, the date of birth and gender of the patient, the date of the colonoscopy, a coded identity of the endoscopist, and the indication and priority of the colonoscopy according to the endoscopist.

Endoscopic information collected included the adequacy of the preparation and cecal intubation and all endoscopic findings. Tumors, as determined to be a likely malignant lesion in the endoscopist's opinion, were recorded as a stand-alone category as “tumors.” Possible important findings were regrouped* a priori* into five hierarchal analytical categories, such that only one finding was assigned to each patient/procedure: tumors, ileocolitis (proctitis, left sided, pancolitis: Crohn's or ulcerative colitis, distal ileitis, colonic or small bowel ulcers, and pseudomembranous or radiation colitis), and polyps ≥ 10 mm were regrouped into a category termed clinically significant lesions. Diverticulosis and a miscellaneous category of pertinent lesions (angiodysplasia, hemorrhoids if no other source of bleeding exists, and rectal strictures) formed individual categories. Other findings such as polyps < 10 mm, hypertrophied anal papillae, melanosis coli, spastic left or a tortuous colon were considered normal or noncontributory findings.

A separate research assistant blinded to the recordings of the study carried out an independent validation of 10% of all collected data.

The Institutional Review Board at the McGill University Health Centre approved the study.

### 2.4. Statistical Analysis

Descriptive statistics were carried out on recorded variables. Means and standard deviations were used to describe continuous variables and proportions with 95% confidence intervals (CI) for categorical variables. Agreement between referring physicians and endoscopists was quantified using a weighted kappa value (with 95% CI) following the Landis-Koch benchmarks [3]. The strength of agreement of the kappa values was characterized as follows: 0 poor; 0–0.20 slight; 0.21–0.40 fair; 0.41–0.60 moderate; 0.61–0.80 substantial; and 0.81–1.00 almost perfect.

Univariable and multivariable analyses using logistic regression were carried out to identify possible associations between patient age, gender, and priority rating and agreement between the referring physician and the endoscopist assessments of priority. For prediction of clinically significant lesions, different multivariable models were created adopting as reference, in turn, each of the different priority ratings (according to both the referring physician and the endoscopist). This method generated multiple ORs for priority ratings. Only significant results are shown and reported with OR and 95% CI. Full detailed results of all the multivariable models are available upon request. A *p* value of 0.05 was considered significant. All analyses were done using SAS software version 9.3 (SAS Institute, Cary, NC, USA).

## 3. Results

### 3.1. Study Population and Procedural Performance

Overall, 853 family medicine or primary care physicians and 30 gastroenterology specialists or surgeons comprised the referring physician population and 8 gastroenterologists performed the study colonoscopies.

Over the 6-month study period, among the 3576 referral requests for colonoscopies, we identified 1230 successive patients with completely filled outpatients referral sheets that comprised the study population (the form was not obligatorily used for every colonoscopy request during this transition period of CTS implementation). As seen in Table 1, the mean patient age was 60.3 ± 12.1 years, and 52.5% were female.

Among all colonoscopies, good or excellent bowel preparations were noted in 86.7%, and the cecum was reached in 95.9% cases. The polyp detection rate was 45.6%, including 20% of total polyp removal which was 10 mm or larger in size (Table 1).

### 3.2. Endoscopic Findings

**Figure 2:**
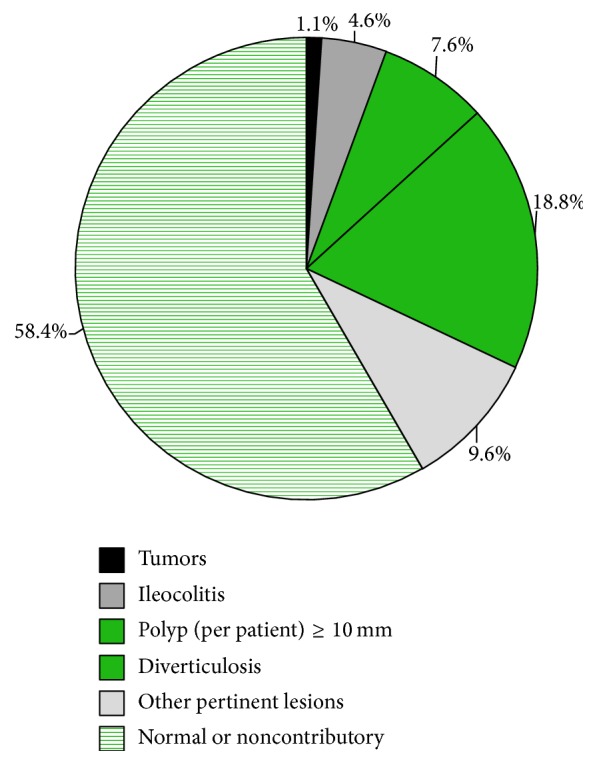
Endoscopic findings *n* = 1230.

Endoscopic findings included tumors (1.1%), polyps per patient ≥ 10 mm (7.6%), ileocolitis (4.6%), and diverticulosis (18.8%). Miscellaneous clinical lesions were noted in 9.6% of cases. Noncontributory findings (including polyps < 10 mm) or a normal colonoscopy were recorded in 58.4% of cases (Figure 2).

### 3.3. Agreement in Priorities and Indications between Referring Physicians and Endoscopists

The assigned priorities and indications, as determined by referring physician and endoscopist, respectively, are listed in Tables 2 and 3. Agreement between referring physician and endoscopist on the 6 triaging priorities for the entire patient population yielded a weighted kappa value of 0.55 (0.51; 0.59), which reflects moderate agreement. Agreement on the 16 indications these priorities are based on was similar, 0.52 (0.48; 0.57).

When looking at the subgroups of patients in whom tumors, ileocolitis, and polyps ≥ 10 mm were found, corresponding weighted kappa values were 0.55 (0.44; 0.67) for the 6 priorities and 0.54 (0.35; 0.73) for the 16 indications.

The agreement on CTS triaging priorities between referring MDs who were primary care physicians and endoscopists yielded a weighted kappa value of 0.57 (0.52; 0.62), reflecting moderate agreement. With regard to agreement between referring MDs who were specialists and endoscopists, the weighted kappa value was fair, 0.30 (0.00; 0.56).

Between-group comparison demonstrated greater proportions of disagreements than agreements for immediate priority P1 (1.0% versus 0.0%, *p* value = 0.01) and semielective priority P3 < 60 days (33.2% versus 25.8%, *p* value = 0.01) but the reverse for P4 (8.2% versus 15.0%, *p* value < 0.01) and P5 (40.1% versus 46.8%, *p* value = 0.04), elective and screening priorities, respectively.

Multivariable analyses identified significant independent predictors of agreement in CTS priority ratings between referring physician and endoscopist. The different predictive models (using different priorities as reference in turn) identified elective priorities P4 with a significant higher level of agreement than urgent priority P2 (OR = 4.57; 1.81; 11.54, P2 as the reference in model) as well as P3 (OR = 2.47; 1.52; 4.00, P3 as the reference in model) and P6 (OR = 2.51; 1.44; 4.39, P6 as the reference in model). P5 also reached a significant higher level of agreement than P2 (OR = 2.93; 1.25; 6.87, P2 as the reference in model) as well as P3 (OR = 1.58; 1.16; 2.16, P3 as the reference in model) and P6 (OR = 1.61; 1.06; 2.45, P6 as the reference in model). Expertise of referring physician categorized as family medicine or GP and gastroenterology or general surgery was not significantly different.

### 3.4. Yield of Priority Selection according to the Referring Physician Priority Rating

Between-group analyses identified patient age (60.2 ± 12.0 versus 74.1 ± 10.6 yrs) and CTS priority 2 (1.7 versus 20.0%) as significantly associated with finding a tumor at colonoscopy (*p* value < 0.01). No stable multivariable model could be constructed for tumor prediction because of the small number of patients having tumors (*n* = 13).

Male gender (54.1% versus 42.6%) and CTS priority 2 (1.5% versus 4.7%) were significantly associated with a finding of tumor, ileocolitis, or polyps ≥ 10 mm in between-group analysis.

Among patients referred because of digestive symptoms, multivariable modeling identified priorities P1 and P2 as significantly more associated with a finding of tumor, ileocolitis, or polyps ≥ 10 mm than priority P4 (P1 and P2: OR = 6.26, 1.2; 33.4, P4 as the reference in model) or P5 (P1 and P2: OR = 4.92, 1.3; 18.7, P5 as the reference in model). Additionally, a priority P6 predicted more such findings than priority P5 (OR = 2.47, 1.07; 5.74, P5 as the reference in model) as well as male gender (OR = 2.04, 1.07; 3.86).

### 3.5. Yield of Priority Selection according to the Endoscopist Priority Rating

The between-group results according to the endoscopists' priority ratings showed patient age (60.2 ± 12.0 versus 74.1 ± 10.6 yrs, *p* value < 0.01) and priorities P2 (3.8 versus 15.4%, *p* value = 0.03) and P3 (21.9 versus 53.9%, *p* value < 0.001) were significantly associated with a finding of tumor.

No stable multivariable model could be constructed for tumor prediction because of the small number of patients having tumors (*n* = 13).

The between-group analyses associated with a finding of tumor, ileocolitis, or polyps ≥ 10 mm are female gender (54.1% versus 42.6%, *p* value = 0.007), CTS priority 2 (3.2% versus 8.6%, *p* value < 0.01), CTS priority 5 (41.8% versus 23.5%, *p* value < 0.01), and CTS priority 6 (13.5% versus 27.2%, *p* value < 0.01).

The series of multivariable models examining the prediction of endoscopic findings of tumor, ileocolitis, or polyps ≥ 10 mm determined significant variables including male gender (OR 1.44, 1.03; 2.03), priorities P1 and P2 that were superior to priorities P3 (P1 and P2: OR 2.14, 1.04; 4.43, P3 as the reference in model), P4 (P1 and P2: OR 2.90, 1.35; 6.23, P4 as the reference in model), and P5 (P1 and P2: OR 4.30, 2.08; 8.88, P5 as the reference in model). Additionally, priority P6 was superior to P5 (P6: OR 3.47, 2.16; 5.59, P5 as the reference in model).

## 4. Discussion 

In the presence of population-based colorectal cancer screening programs, public health authorities must secure equitable access to a timely endoscopy both to screening patients and to those with digestive symptoms. It therefore becomes primordial to establish an appropriate framework to manage access and waiting times for colonoscopy.

In 2006, The New York City Department of Health and Mental Hygiene published a colorectal cancer screening toolkit named “A Practical Guide to Increasing Screening Colonoscopy” in which authorities distributed a single page “General Referral for Colonoscopy” sheet [4]. While the sheet contained five broad categories of symptoms for referral for a colonoscopy with a component that allowed the flagging of patients who required a prior consultation because of comorbidities (e.g., hypertension, heart failure, and pulmonary disease), no associated target times to undergo the procedure were identified.

In preparation for full implementation of the Quebec colorectal cancer screening program, an evidence-based CTS was developed to manage access to colonoscopy in an optimized and equitable manner. The CTS was constructed based on colonoscopy clinical guidelines issued by the Quebec Ministry of Health (with only approved indications for colonoscopy included in the CTS, after a series of literature reviews) [5] and published waiting time recommendations by the Canadian Association of Gastroenterology [2]. The CTS can be downloaded from the Quebec Ministry of Health website and is sent to endoscopy units using existing resources (fax machines and regular mail), thereby not imposing additional costs since it replaces a standard request for consultation form.

The current retrospective study consisted of a consecutive sample of outpatients referred to the MGH site. After exclusion of missing data, we identified 1230 distinct outpatients referral sheets. Missing data were partly explained by the recent introduction of the CTS and its nonobligatory nature at the time. It is important to note that the demographics of the population with missing data were similar to those of the included patients (average age 60.3; 47.5% men). Indeed, a study conducted in the same endoscopy unit (MUHC-MGH) from April 1, 2013, to April 30, 2014, looked at the information on 2730 patients and found a proportion of 50.1% men and a mean age of 60.4 years [6]. The data analyzed in our study therefore are likely to be a representative sample of the patient population in the unit without obvious reason for or presence of selection bias. The very large number of referring physicians included in the study minimizes the likelihood of selection bias in this physician population.

With respect to endoscopic findings, tumors, ileocolitis, and polyps ≥ 10 mm were included in the inferential analyses to assess the yield of the priority ratings given their importance as markers to finding advanced neoplasia. All patients with documented findings of tumors had a diagnosis of adenocarcinoma confirmed on subsequent pathological analysis. While the clinical relevance of tumors and ileocolitis is obvious, polyps ≥ 10 mm were included as they imply an earlier subsequent endoscopic surveillance interval [7, 8]. Inclusion of diminutive polyps in the absence of histological analysis, which was unavailable, was not considered as it would introduce much more noise in the analysis, with minimal true clinical significance since recent data suggest that patients with sole diminutive polyps may be at even lower risk of subsequent neoplasia development. Although findings of diverticulosis were recorded, they were not included in the analyses because they tend to be incidental findings in the great majority of patients [9]. Nonetheless, it is clear that this assembling of findings was an arbitrary choice for the purposes of analysis.

Our sample did not include sufficient numbers of patients without symptoms to create a stable multivariable model. Patients with symptoms represent the population that motivated the creation of the hierarchal set of indications appearing in the CTS. The assigned CTS priority ratings and their predictive ability varied according to whether they were assigned by the referring physician or the endoscopist; urgent priority ratings (P1 or P2, colonoscopy within 14 days) were predictors of tumors and of clinically significant findings as opposed to more elective ratings (P4 (least urgent priority rating for patients with symptoms) or P5 (average-risk screening patients)); similar results were noted for the surveillance priority P6 compared to the screening priority P5. Internal validation of our analyses is supported in our cohort by the findings that older individuals were most at risk of developing a tumor, and male gender was significantly associated with clinical significant lesions, as both are widely recognized in the literature [10].

Priority 1 referrals were very few, limiting any statistical inference for this subgroup; indeed, the corresponding patients are suffering from acute lower gastrointestinal hemorrhage and need assessment within 24 hours. They are often seen in the emergency room as soon as possible and bypass the entire usual colonoscopy referring process.

The improved yield of the surveillance priority (P6) with regard to clinically significant findings at colonoscopy as compared to the average-risk priority (P5) can be explained by the sample including a subgroup of patients at a particular high risk of recurring polyps in follow-up [7, 11].

Additional data are required to better understand the comparative predictive ability of priority ratings P3 versus P4 and P4 versus P5. Alternately, a grouping of categories could be assessed, but we based our classification on the past CAG guideline document on waiting times [2].

As the complexity of colorectal cancer biology renders accurate prediction based on symptoms difficult, it is challenging to do studies measuring the predictability of colorectal cancer based on symptoms [12]. Nonetheless, these results appear to validate, at least in a broad fashion, the adopted CTS hierarchal priority rating scheme and sew the basis for further refinement and validation of the CTS tool as it is deployed in Quebec.

Only moderate agreement was found between referring physicians and endoscopists (weighted kappa value of 0.55 (0.51; 0.59)) for the assignment of CTS priority ratings. Although the finding is likely generalizable considering the sample of 853 referring physicians, mostly in primary practice, reasons for this moderate agreement are likely multifactorial. First, the condition of the patient may have evolved between the time of the consultation with the referring physician and the colonoscopy procedure. Indeed, based on the recorded wait times of a random sampling of 230 colonoscopies performed during the study period, the average elapsed duration between the date we received the request from the referring physician and the assessment by the endoscopist on the day of the procedure was 5.79 months. Secondly, an accurate identification of qualifying symptoms and subsequent referral for colonoscopy may depend on the thoroughness of clinical assessment of the presenting GI symptoms with referring practitioners emphasizing different symptoms than endoscopists, resulting in disparate perceived indications for colonoscopy. Patients at different times may emphasize different symptoms. The observed higher agreement for less urgent priorities may reflect the consistency in patients reporting symptoms that have been present for long periods of time.

Finally, patients or physicians' reporting of symptoms may have been altered due to perceived long wait times for colonoscopy [13]. Indeed, patients may have misreported symptoms to their referring physicians to gain faster access to a colonoscopy appointment or perhaps referring physicians, cognizant of long waiting times for colonoscopy, willingly or unwillingly gamed the system in order to get an earlier appointment for their patients. Moving forward, some educational interventions among referring physicians at professional societies annual meetings and workshops can promote a more uniform understanding of the rationale and utility of the CTS and hopefully lead to better agreement on the need and timeliness of a colonoscopy. Finally, although there were no differences in patient characteristics according to inclusion/exclusion in the study sample, referring physicians who completed the CTS forms may be different from those who did not complete the forms, despite the very large sampling of referring physicians. Whether these possible differences affect our findings will need to be determined in a follow-up study. Such a study would also determine how often patients are actually seen for colonoscopy within the recommended time frames.

One of the strengths of the colonoscopy triage sheet is its potential to standardize the referral process and help manage waiting lists in an efficient and equitable manner across the province. The external validity in other provinces or outside Canada would need to be examined. In follow-up to this initial assessment of the CTS, a decision support tool comprising a lexicon explaining why each indication is associated with a high or low priority has been posted on the website of the Quebec Health Ministry and is being disseminated among general practitioners and specialists alike.

## 5. Conclusion

In conclusion, the agreement on triaging priorities between referring physician and endoscopist was moderate. Less urgent priority ratings (P4 and P5) were associated with higher agreement between referring physician and endoscopist compared to the urgent priority ratings. Increasing age, male gender, and urgent CTS priorities were significantly associated with finding a tumor. The proposed hierarchical priority setting among urgent and elective settings appeared to have been validated by the findings of clinically significant lesions at endoscopy.

In order to optimize the utility of the CTS, physician education is now required to improve CTS priority rating assignment among referring physicians to further refine the allocation of symptoms to less urgent priorities, especially for P3 versus P4 ratings.

## Figures and Tables

**Table 1:** Patient population demographics.

Patient characteristics	(%; 95% CI) or mean ± (std) (*n* = 1230)
Female (%)	52.5 (49.7; 55.3)
Age (years)	60.3 ± 12.1
Bowel preparation score (excellent/good) (%)	86.7 (84.4; 89.1)
Cecal intubation (%)	95.9 (94.7; 97.0)
Polyps (all polyps found) (%)	45.6 (42.7; 48.6)
Polyps ≤ 5 mm (% of all polyps)	47.6 (43.1; 52.0)
Polyps (5–9.9 mm) (% of all polyps)	32.5 (28.4; 36.7)
Polyps ≥ 10 mm (% of all polyps)	20.0 (16.4; 23.5)
Polyps ≥ 10 mm (% per patient)	7.6 (6.2; 9.1)
Mean number of polyps per patient	2.3 ± 2.1 (median: 2.0; Q1: 1.0; Q3: 3.0)

Note: there are 19 indications as indications 9 and 16 were removed.

**Table 2:** Priority by referring physician and endoscopist for all findings.

	Referring physician priority, percentage of patients with this priority (%; 95% CI) or mean (std) (*n* = 1053)^*∗*^	Endoscopist priority, percentage of patients with this priority (%; 95% CI) or mean (std) (*n* = 1053)^*∗*^
Priority 01, immediate	0.3 (0.0; 0.6)	0.0^*∗∗*^
Priority 02, urgent ≤ 14 days	1.9 (1.1; 2.7)	3.9 (2.7; 5.1)
Priority 03, semielective ≤ 60 days	28.3 (25.6; 31.0)	21.8 (19.3; 24.2)
Priority 04, elective ≤ 6 months	12.7 (10.7; 14.7)	20.3 (17.9; 22.8)
Priority 05, screening	44.5 (41.5; 47.6)	41.3 (38.3; 44.3)
Priority 06, surveillance	12.3 (10.3; 14.2)	12.7 (10.7; 14.7)
*Weighted kappa*	*0.55 (0.51; 0.59)*	

^*∗*^177 missing priorities (only indications provided on the CTS form).

^*∗∗*^No formal priority rating carried out in this group by the endoscopists as none were colonoscoped as outpatients; all were referred through the emergency room or as inpatients.

**Table 3:** Indication by referring physician and endoscopist for all findings.

	Indication referral (%; 95% CI) or mean (std) (*n* = 1045)^*∗*^	Endoscopist indication (%; 95% CI) or mean (std) (*n* = 1045)^*∗*^
Indication 01 or 02, immediate or urgent ≤ 14 days	2.0 (1.2; 2.9)	3.6 (2.5; 4.8)^*∗∗*^
Indication 03, semielective ≤ 60 days	1.4 (0.7; 2.2)	0.3 (0.0; 0.6)
Indication 04, semielective ≤ 60 days	7.2 (5.6; 8.7)	8.5 (6.8; 10.2)
Indication 05, semielective ≤ 60 days	2.2 (1.3; 3.1)	1.6 (0.9; 2.4)
Indication 06, semielective ≤ 60 days	8.2 (6.6; 9.9)	7.1 (5.5; 8.6)
Indication 07, semielective ≤ 60 days	7.6 (6.0; 9.2)	3.9 (2.7; 5.1)
Indication 08 or 09, elective ≤ 6 months	11.4 (9.5; 13.3)	18.8 (16.4; 21.1)
Indication 10, elective ≤ 6 months	1.5 (0.8; 2.3)	1.4 (0.7; 2.2)
Indication 11, screening	36.7 (33.7; 39.6)	37.5 (34.6; 40.5)
Indication 12, elective ≤ 6 months	8.0 (6.4; 9.7)	3.8 (2.7; 5.0)
Indications 13 to 16, follow-up	13.8 (11.7; 15.9)	13.1 (11.1; 15.2)
*Weighted kappa*	*0.52 (0.48; 0.57)*	

^*∗*^185 missing indications (only indications provided on the CTS form).

^*∗∗*^Includes no endoscopist indication corresponding to a P1 priority rating as no such patients were colonoscoped as outpatients; all were referred through the emergency room or as inpatients.

## References

[1] Canadian Cancer Society's Advisory Committee on Cancer Statistics (2014). *Canadian Cancer Statistics 2014*.

[2] Paterson W. G., Depew W. T., Paré P. (2006). Canadian consensus on medically acceptable wait times for digestive health care. *Canadian Journal of Gastroenterology*.

[3] Landis J. R., Koch G. G. (1977). The measurement of observer agreement for categorical data. *Biometrics*.

[4] Cancer Prevention and Control Program Bureau of Chronic Disease Prevention and Control *A Practical Guide to Increasing Screening Colonoscopy*.

[5] http://msssa4.msss.gouv.qc.ca/intra/formres.nsf/9d7020958f686e8a85256e4500715a8f/05d305da385afcd2852579f30053d4b6?OpenDocument.

[6] Kherad O., Restellini S., Martel M., Barboza L. M., Barkun A. N. (2015). Which is the best product to use when preparing a colon?. *Canadian Journal of Gastroenterology*.

[7] Leddin D., Enns R., Hilsden R. (2013). Colorectal cancer surveillance after index colonoscopy: guidance from the Canadian Association of Gastroenterology. *Canadian Journal of Gastroenterology*.

[8] Lieberman D. A., Rex D. K., Winawer S. J., Giardiello F. M., Johnson D. A., Levin T. R. (2012). Guidelines for colonoscopy surveillance after screening and polypectomy: a consensus update by the us multi-society task force on colorectal cancer. *Gastroenterology*.

[9] Templeton A. W., Strate L. L. (2013). Updates in diverticular disease. *Current Gastroenterology Reports*.

[10] Wei E. K., Giovannucci E., Wu K. (2004). Comparison of risk factors for colon and rectal cancer. *International Journal of Cancer*.

[11] Rex D. K., Schoenfeld P. S., Cohen J. (2015). Quality indicators for colonoscopy. *Gastrointestinal Endoscopy*.

[12] Adelstein B.-A., Macaskill P., Chan S. F., Katelaris P. H., Irwig L. (2011). Most bowel cancer symptoms do not indicate colorectal cancer and polyps: a systematic review. *BMC Gastroenterology*.

[13] Paterson W. G., Barkun A. N., Hopman W. M. (2010). Wait times for gastroenterology consultation in Canada: the patients' perspective. *Canadian Journal of Gastroenterology*.

